# Rice Paddy Nitrospirae Carry and Express Genes Related to Sulfate Respiration: Proposal of the New Genus “Candidatus Sulfobium”

**DOI:** 10.1128/AEM.02224-17

**Published:** 2018-02-14

**Authors:** Sarah Zecchin, Ralf C. Mueller, Jana Seifert, Ulrich Stingl, Karthik Anantharaman, Martin von Bergen, Lucia Cavalca, Michael Pester

**Affiliations:** a Department of Biology, University of Constance, Constance, Germany; b Dipartimento di Scienze per gli Alimenti, la Nutrizione e l'Ambiente (DeFENS), Università degli Studi di Milano, Milan, Italy; c Institute of Animal Science, Hohenheim University, Stuttgart, Germany; d University of Florida, UF/IFAS, Department for Microbiology and Cell Science, Fort Lauderdale Research and Education Center, Davie, Florida, USA; e Department of Earth and Planetary Science, University of California, Berkeley, California, USA; f Helmholtz Centre for Environmental Research—UFZ, Department of Molecular Systems Biology, Leipzig, Germany; g Department of Microorganisms, Leibniz Institute DSMZ—German Collection of Microorganisms and Cell Cultures, Braunschweig, Germany; University of Bayreuth

**Keywords:** sulfate-reducing microorganisms, rice paddies, gypsum fertilization, *dsrAB* genes, Nitrospirae

## Abstract

Nitrospirae spp. distantly related to thermophilic, sulfate-reducing Thermodesulfovibrio species are regularly observed in environmental surveys of anoxic marine and freshwater habitats. Here we present a metaproteogenomic analysis of Nitrospirae bacterium Nbg-4 as a representative of this clade. Its genome was assembled from replicated metagenomes of rice paddy soil that was used to grow rice in the presence and absence of gypsum (CaSO_4_·2H_2_O). Nbg-4 encoded the full pathway of dissimilatory sulfate reduction and showed expression of this pathway in gypsum-amended anoxic bulk soil as revealed by parallel metaproteomics. In addition, Nbg-4 encoded the full pathway of dissimilatory nitrate reduction to ammonia (DNRA), with expression of its first step being detected in bulk soil without gypsum amendment. The relative abundances of Nbg-4 were similar under both treatments, indicating that Nbg-4 maintained stable populations while shifting its energy metabolism. Whether Nbg-4 is a strict sulfate reducer or can couple sulfur oxidation to DNRA by operating the pathway of dissimilatory sulfate reduction in reverse could not be resolved. Further genome reconstruction revealed the potential to utilize butyrate, formate, H_2_, or acetate as an electron donor; the Wood-Ljungdahl pathway was expressed under both treatments. Comparison to publicly available Nitrospirae genome bins revealed the pathway for dissimilatory sulfate reduction also in related Nitrospirae recovered from groundwater. Subsequent phylogenomics showed that such microorganisms form a novel genus within the Nitrospirae, with Nbg-4 as a representative species. Based on the widespread occurrence of this novel genus, we propose for Nbg-4 the name “Candidatus Sulfobium mesophilum,” gen. nov., sp. nov.

**IMPORTANCE** Rice paddies are indispensable for the food supply but are a major source of the greenhouse gas methane. If it were not counterbalanced by cryptic sulfur cycling, methane emission from rice paddy fields would be even higher. However, the microorganisms involved in this sulfur cycling are little understood. By using an environmental systems biology approach with Italian rice paddy soil, we could retrieve the population genome of a novel member of the phylum Nitrospirae. This microorganism encoded the full pathway of dissimilatory sulfate reduction and expressed it in anoxic paddy soil under sulfate-enriched conditions. Phylogenomics and comparison to the results of environmental surveys showed that such microorganisms are actually widespread in freshwater and marine environments. At the same time, they represent an undiscovered genus within the little-explored phylum Nitrospirae. Our results will be important for the design of enrichment strategies and postgenomic studies to further understanding of the contribution of these novel Nitrospirae spp. to the global sulfur cycle.

## INTRODUCTION

Sulfate-reducing microorganisms (SRM) are regularly observed in rice paddy fields (1–8). Despite the prevailing low sulfate concentrations in this habitat (lower micromolar range [9, 10]), the rice rhizosphere and bulk soil are characterized by high sulfate reduction rates, comparable to those in marine surface sediments (11). This observation is explained by a cryptic sulfur cycle. Here, the small sulfate pool is rapidly reduced to sulfide, but the latter is also rapidly reoxidized to sulfate, keeping a highly active sulfur cycle running (10–13). This cryptic sulfur cycle can occur at oxic-anoxic interfaces, such as rice roots, but apparently runs also in completely anoxic bulk soil (10). Under the latter conditions, reduced sulfur species may be reoxidized with the help of iron minerals or redox-active parts of humic material, such as quinone moieties, as shown for other freshwater habitats (14–16).

The ability to perform dissimilatory sulfate reduction is most widespread among members of the Deltaproteobacteria and Firmicutes (17). Additional and exclusively thermophilic sulfate reducers are affiliated with the archaeal phyla Euryarchaeota and Crenarchaeota and the bacterial phyla Thermodesulfobacteria and Nitrospirae (17, 18). The only known SRM in the phylum Nitrospirae are bacteria belonging to the genus Thermodesulfovibrio (19–23). All described species of this genus are thermophilic; their common metabolic properties comprise the reduction of sulfate, thiosulfate, and, in some cases, sulfite with a limited range of electron donors. These include pyruvate and lactate, which are incompletely oxidized to acetate, or H_2_ and formate in a background of acetate as an auxiliary carbon source. The inability to grow autotrophically and the incomplete oxidation of organic substrates to acetate are characteristic features of this genus. Alternative electron acceptors used by Thermodesulfovibrio spp. are Fe(III) and, in the case of Thermodesulfovibrio islandicus DSM 12570, nitrate (19–23).

In addition to the genus Thermodesulfovibrio, the phylum Nitrospirae currently encompasses the genera Nitrospira and Leptospirillum (24). Nitrospira spp. are known to have a versatile metabolism ranging from chemolithoautotrophic ammonia, nitrite, or hydrogen oxidation coupled to oxygen respiration to formate-driven nitrate respiration to nitrite (reviewed in reference 25). Leptospirillum spp. are described as iron oxidizers (24). A group of still uncultured Nitrospirae, which form a sister clade to the genus Thermodesulfovibrio, is represented by magnetotactic bacteria belonging to the putative genera “Candidatus Magnetobacterium” (26–28), “Candidatus Thermomagnetovibrio” (29), “Candidatus Magnetoovum” (30, 31), and “Candidatus Magnetominusculus” (32). These microorganisms are typically encountered at the oxic-anoxic interfaces of sediments but were also enriched from the water of hot springs (33). The observation of sulfur-rich inclusions in the cells of “Candidatus Magnetobacterium bavaricum” (27), “Candidatus Magnetoovum chiemensis” (31), and “Candidatus Magnetoovum mohavensis” (30), the presence of sulfur metabolism genes in the genomes of the former two species (31), and their predominant occurrence at oxic-anoxic interfaces led to the hypothesis that these microorganisms could be involved in sulfur oxidation (27, 31, 33).

All SRM encode the canonical pathway of dissimilatory sulfate reduction, an intracellular process that involves an eight-electron reduction of sulfate to sulfide. This pathway proceeds through the enzymes sulfate adenylyltransferase (Sat), adenylyl phosphosulfate reductase (Apr), dissimilatory sulfite reductase (Dsr), and the sulfide-releasing DsrC (34). In addition, the complexes QmoAB(C) and DsrMK(JOP) are important in transferring reducing equivalents toward the pathway of sulfate reduction (35). The only known exceptions to this rule are anaerobic methanotrophic (ANME) archaea—archaea that anaerobically oxidize methane by a yet unresolved mechanism of sulfate reduction to zero-valent sulfur (36).

The two different subunits of the heterotetrameric dissimilatory sulfite reductase Dsr are encoded by the paralogous genes *dsrA* and *dsrB*, which are frequently used as functional phylogenetic markers for SRM (37). The phylogeny of reductive bacterial-type DsrAB is subdivided into the Deltaproteobacteria, Firmicutes, environmental, and Nitrospirae superclusters (37). DsrAB sequences affiliated with the Nitrospirae supercluster have been found predominantly in freshwater and soil environments and, to a smaller extent, in marine, industrial, or high-temperature habitats (37). Intriguingly, these sequences have also been detected in Italian (10) and Chinese (4, 8) rice paddy soils, but the detailed phylogenetic affiliation of these *dsrAB*-carrying microorganisms and their possible involvement in rice paddy sulfur cycling have remained unclear.

In this study, the draft genome of a novel and putatively sulfate reducing species belonging to the phylum Nitrospirae has been obtained from a metagenome survey of rice paddy soil. We present its metabolic potential and phylogeny as reconstructed from its genome, and we compare this to Nitrospirae genome bins recently recovered from metagenome studies of groundwater habitats. To support our conclusions, we present protein expression patterns of this novel Nitrospirae species as inferred by a metaproteome analysis of rice paddy soil.

(This article was submitted to an online preprint archive [38].)

## RESULTS

### A Nitrospirae genome from rice paddy soil.

**FIG 1:**
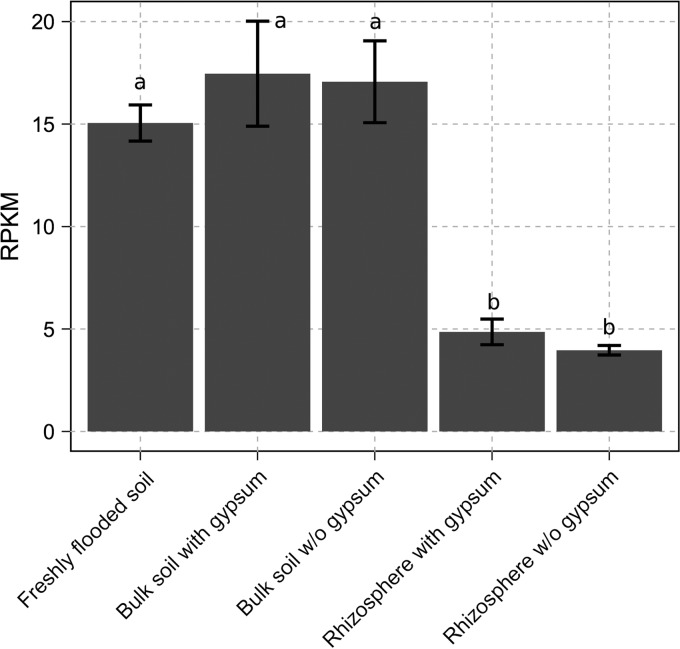
Average relative abundances (±1 standard deviation) of Nitrospirae bacterium Nbg-4 in differently treated soil habitats as inferred from the RPKM (reads per kilobase of scaffold per million reads) values of its longest scaffold. Significant differences are indicated by different letters above the bars and were inferred by a two-way ANOVA and a *post hoc* Tukey test (*P* < 0.001). w/o, without.

We used a metagenomics approach to identify novel microorganisms involved in rice paddy sulfur cycling. For this purpose, replicated metagenomes (see Table S1 in the supplemental material) were sequenced from bulk and rhizosphere soils of rice plants, which were grown to their late vegetative phase either in gypsum (CaSO_4_·2H_2_O)-amended or unamended (control) soils. In addition, metagenomes from freshly flooded and unplanted soils were analyzed. Among the 159 population genome bins that could be retrieved, Nitrospirae genome bin Nbg-4 was outstanding in that it encoded *dsrAB*, was of high quality with ≤2% residual contamination, showed no strain heterogeneity, and had an estimated genome completeness of 75% (Table 1). The relative abundance of Nbg-4 was highest in the bulk soils (averaging 17 reads per kilobase of scaffold per million reads [RPKM]) and roughly three times lower in rhizosphere soils (Fig. 1). Two-way analysis of variance (ANOVA) showed that the soil compartment had a significant effect on the relative abundance of Nbg-4 (F_2,14_ = 36.16; *P* < 0.001), while gypsum amendment (F_1,14_ = 0.17; *P* = 0.69) and the interaction of the soil compartment and gypsum amendment (F_1,12_ = 0.03; *P* = 0.87) remained insignificant. To estimate the index of replication (iRep) (39) of Nbg-4, single reads of metagenomic replicates were combined per soil habitat to achieve sufficient coverage. This analysis indicated that roughly three-quarters of the population were replicating their genomes in freshly flooded soils, while roughly one-third replicated their genomes in bulk soils after 58 to 59 days of incubation irrespective of gypsum treatment (Table 1). For rhizosphere soils, the coverage was insufficient to perform an iRep analysis.

**TABLE 1:** Characteristics of the obtained draft genome of Nitrospirae bacterium Nbg-4

Characteristic	Value for Nitrospirae bacterium Nbg-4
Genome feature	
Chromosome size (Mbp)	2.77
GC content (%)	49
No. of scaffolds	151
No. of CDS^*a*^	2,855
Avg CDS length (bp)	855
Protein-coding density (%)	87
No. of rRNA genes	1
No. of tRNA genes	21
CheckM analysis	
Completeness (%)	75.5
Contamination (%)	2.0
Strain heterogeneity (%)	0.0
iRep analysis	
In initial soil	1.73
In bulk soil without gypsum	1.34
In bulk soil with gypsum	1.31

a CDS, coding DNA sequences.

### Reconstruction of a dissimilatory sulfur metabolism.

**FIG 2:**
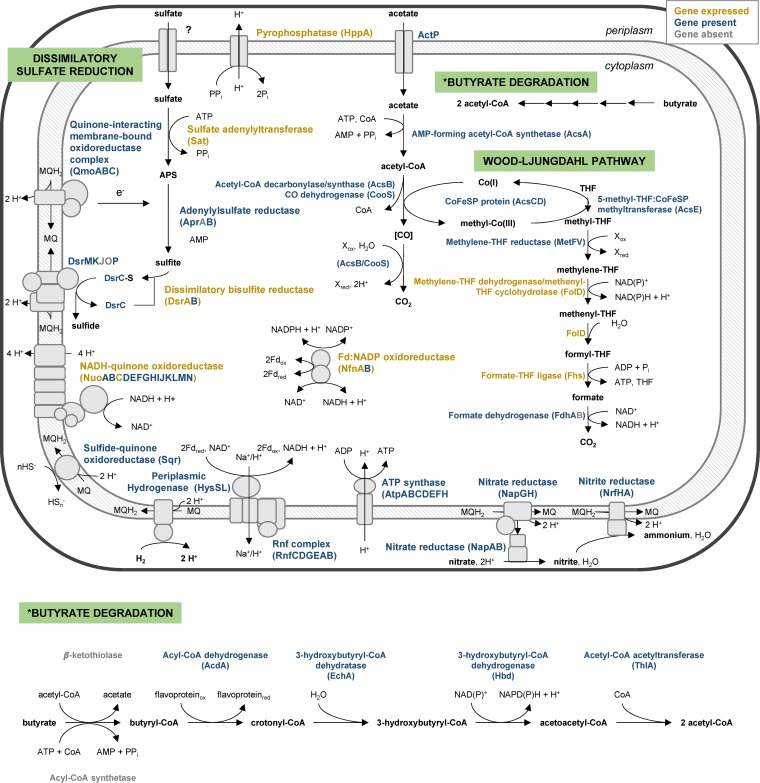
Schematic view of reconstructed energy metabolism pathways in Nitrospirae bacterium Nbg-4. The expression of proteins in bulk soil treated with gypsum, as revealed by metaproteomics, is indicated by color. Protein expression in other soil habitats and in soils with different treatments is given in Table S2.

The complete dissimilatory sulfate reduction pathway was recovered for Nbg-4 (Fig. 2). Besides genes encoding Sat and the beta subunit of Apr, which catalyze the activation of sulfate and its concomitant reduction to sulfite, respectively, genes for DsrAB and DsrC, which reduce sulfite further to sulfide, could also be detected. *aprA* was missing, probably because of an assembly break in the scaffold after *aprB* (typically, *aprA* is downstream of *aprB*). In addition, genes encoding the QmoABC and DsrMK complexes, which couple quinol reduction to electron transfer to AprAB and DsrC, respectively, were detected. Thermodesulfovibrio spp. possess, in addition to the DsrMK module, the DsrJOP module; together, these modules form the membrane-bound electron-transferring complex DsrMKJOP (23, 35). Since *dsrMK* were located at the end of one scaffold in Nbg-4, and another scaffold started with a long fragment of *dsrP*, it is likely that Nbg-4 also encodes a complete DsrMKJOP complex. In addition, the presence of *dsrD* directly adjacent to *dsrAB* was detected. DsrD is a small protein of putative regulatory function present in all sulfate reducers (40); it is sporadically encountered in the genomes of sulfide- and sulfur-oxidizing bacteria (41). In addition, *dsrN* and *dsrT*, typical genes of the *dsr* operon in sulfate reducers and sulfur-oxidizing green sulfur bacteria (40, 42), and *hppA*, which codes for a membrane-bound and proton-translocating pyrophosphatase to pull, e.g., the energy-demanding reaction of Sat, were detected (see Table S2 in the supplemental material). With the exception of a membrane-bound sulfide-quinone oxidoreductase (Sqr) (Table S2), no genes that are essential for the oxidative sulfur metabolism of chemolithotrophic sulfur oxidizers (42–44) were detected.

All soil samples that were used for metagenome sequencing were also analyzed for their metaproteome. In bulk soil treated with gypsum, a search against Nbg-4-encoded proteins identified peptides specific for Sat and DsrA, essential components of the first and last step of sulfate reduction, respectively (Fig. 2). Peptides specific for Nbg-4 DsrA were also detected in rhizosphere soil treated with gypsum but at a lower intensity (label-free quantification [LFQ] value in bulk soil, 1.20 × 10^8^; LFQ in rhizosphere soil, 6.05 × 10^4^). In contrast, no peptides matching Nbg-4 sulfur metabolism proteins were detected in control soils without gypsum, neither in the bulk soil nor in the rhizosphere (Table S2). The fragmented recovery of proteins involved in dissimilatory sulfate reduction is certainly a result of the low coverage of the proteome of a single microbial population in the background of the whole soil metaproteome.

**FIG 3:**
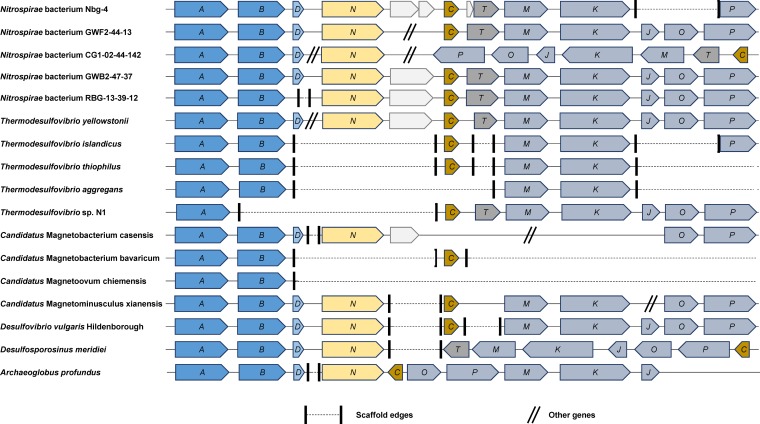
Organization and synteny of the *dsr* operon in Nitrospirae bacterium Nbg-4 with those in other *dsrAB*-carrying members of the phylum Nitrospirae. In addition, data for typical representatives of known sulfate-reducing microorganisms within the Deltaproteobacteria (Desulfovibrio vulgaris Hildenborough), Firmicutes (Desulfosporosinus meridiei), and Archaea (Archaeoglobus profundus) are shown.

Based on the recovery of the dissimilatory sulfate reduction pathway in Nbg-4, NCBI's sequence repositories were searched for additional *dsrAB*-carrying Nitrospirae genome bins of high assembly quality. This analysis identified 14 additional bins recovered from metagenomes: 3 from aquifer sediments (45), 9 from aquifer groundwater (45), and 2 from deep subsurface water (46) (see Table S3 in the supplemental material). In-depth analysis of four bins that represent the three additional habitat types revealed the presence not only of *dsrAB* but also of the complete *dsr* operon, including *dsrC*, *dsrD*, *dsrN*, *dsrT*, and *dsrMKJOP*, which were all in synteny with the respective genes of Nbg-4 (Fig. 3). Only Nitrospirae bacterium CG1-02-44-142, recovered from deep subsurface water, had an inversion of *dsrC*, *dsrT*, and *dsrMKJOP* on its genome. Interestingly, all other components of the dissimilatory sulfate reduction pathway, including *sat*, *aprBA*, *qmoABC*, and *hppA*, were also carried on these Nitrospirae genome bins, either completely or partially, depending on the assembly breaks of the respective scaffolds (Table 2).

**TABLE 2:** Locus tags of genes involved in a dissimilatory sulfur metabolism in Nitrospirae bacterium Nbg-4, related *dsrAB*-carrying Nitrospirae recovered from groundwater metagenomes,^*a*^ and Thermodesulfovibrio yellowstonii

Gene involved in a dissimilatory sulfur metabolism	Locus tag in:					
	Nitrospirae bacterium:					Thermodesulfovibrio yellowstonii (THEYE)
	Nbg-4	GWF2-44-13 (A2X54)	CG1-02-44-142 (AUJ60)	GWB2-47-37 (A2X55)	RBG-13-39-12 (A2Y97)	
*dsrA*	480011	05135	04265	01500	05490	A1994
*dsrB*	480010	05130	04260	01495	05485	A1995
*dsrD*	480009	05125	04255	01490		A1996
*dsrN*	480008	05120	09835	01485	05450	A0001
*dsrC*	480005	00165	04175	01475	05445	A0003
*dsrT*	480003	00170	04180	01470	05440	A0004
*dsrM*	480002	00175	04185	01465	05435	A0005
*dsrK*	480001	00180	04190	01460	05430	A0006
*dsrJ*		00185	04195	01455	05425	A0007
*dsrO*		00190	04200	01450	05420	A0008
*dsrP*	1080008	00195	0425	01445	05415	A0009
*aprA*		02100		02795	02630	A1832
*aprB*	690001	02105		02800	02635	A1833
*sat*	690002	02110	03990	02805	02645	A1835
*hppA*	30083	02080	08585	02770	02470	
*qmoA*	30087	02095	08565	02790	02455	A1831
*qmoB*	30086	02090	08570	02785	02460	A1830
*qmoC*	30085	02085	08575	02780	02465	A1829

a See references 45 and 46.

### Nitrate reduction as an alternative respiratory metabolism.

Nbg-4 also carried a full set of genes necessary for dissimilatory nitrate reduction to ammonia (DNRA) (Fig. 2). DNRA is employed by members of the genera Thermodesulfovibrio, Desulfovibrio, Desulfobulbus, Desulfobacterium, and Desulfotomaculum as an alternative respiratory pathway in the absence of sulfate (40). The first step of DNRA is the reduction of nitrate to nitrite. To perform this step, Nbg-4 contains a periplasmic nitrate reductase, NapA, that forms a soluble complex with cytochrome *c*-containing NapB and couples electron transfer from the quinone pool by the membrane-associated quinol dehydrogenase module formed by NapGH (Table S2). In Nbg-4, the *nap* operon lacks a gene encoding NapC, which is a proposed electron-transferring, membrane-associated protein typically observed in DNRA-performing SRM. The lack of NapC resembles the situation in Wolinella succinogenes, which also lacks this protein but is able to perform DNRA (47). The second step of DNRA employs a six-electron transfer to reduce nitrite to ammonia. In Nbg-4, this step might be catalyzed by the membrane-bound nitrite reductase complex formed by NrfA, a periplasmic nitrite reductase, and NrfH, a membrane-associated quinol dehydrogenase that delivers electrons to NrfA. Screening of the metaproteomes for DNRA-related proteins of Nbg-4 identified peptides specific for NapA and NapG in bulk soils (LFQ, 1.35 × 10^7^ and 4.87 × 10^3^, respectively) without gypsum treatment. The lack of peptides specific for proteins involved in the second step of DNRA could, again, be due to the fragmented recovery of the metaproteome. However, specific expression of the first DNRA step only, without further conversion of the nitrite produced to ammonium, cannot be excluded. No peptides of DNRA-related proteins were detected in bulk soil treated with gypsum or in the rhizosphere samples, irrespective of gypsum treatment (Table S2).

### Genetic potential for complete oxidation of organic matter to CO_2_.

The genome of Nbg-4 encoded the capacity for complete oxidation of acetate to CO_2_. This included the acetate transporter ActP, activation of acetate to acetyl coenzyme A (acetyl-CoA) by an AMP-forming acetyl-CoA synthetase (AcsA), and the complete Wood-Ljungdahl pathway (Fig. 2; Table S2). Peptides specific for several of these enzymes could be detected with higher signal intensities in the bulk soil (LFQ, 3.55 × 10^8^ to 5.20 × 10^8^) as in the rhizosphere (LFQ, 2.11 × 10^4^ to 1.69 × 10^6^), whereas gypsum treatment had no apparent effect (Table S2). The Wood-Ljungdahl pathway included at the end of its methyl branch a formate dehydrogenase, which provides Nbg-4 with the potential to utilize formate as an electron donor. In addition, a periplasm-oriented, membrane-bound [NiFeSe] hydrogenase (HysLS), which connects to the quinone pool in the membrane, was detected (Fig. 2). However, no peptides related to either of these two enzyme complexes could be detected (Table S2). Furthermore, the potential for butyrate degradation via β-oxidation was encoded. Except for the activation step of butyrate to butyryl-CoA, all genes encoding the necessary enzymes were recovered (Fig. 2). Peptides that match Nbg-4 enzymes involved in butyrate degradation were detected in rhizosphere but not in bulk soil metaproteomes (LFQ, 4.18 ×10^4^ to 2.66 × 10^6^ [Table S2]).

In addition to the H^+^-translocating quinol reductase complexes mentioned above for the sulfate and nitrate reduction pathways, coupling of electron transfer to energy conservation could be mediated in Nbg-4 by an electron-bifurcating ferredoxin-NADP oxidoreductase (NfnAB), an H^+^/Na^+^-pumping Rnf complex (RnfCDGEAB), and an NADH-quinone oxidoreductase (respiratory complex I, NuoABCDEFGHIJKLMN) (35). In addition, the full set of genes encoding the ATP synthase was identified (AtpABCDEFHI) (Fig. 2). Peptides specific for each of these Nbg-4 enzyme complexes were identified in the various bulk and rhizosphere soil metaproteomes (Table S2), indicating their active roles in electron transfer and energy conservation.

### Phylogenetic affiliation of the Nitrospirae genome bin Nbg-4.

**FIG 4:**
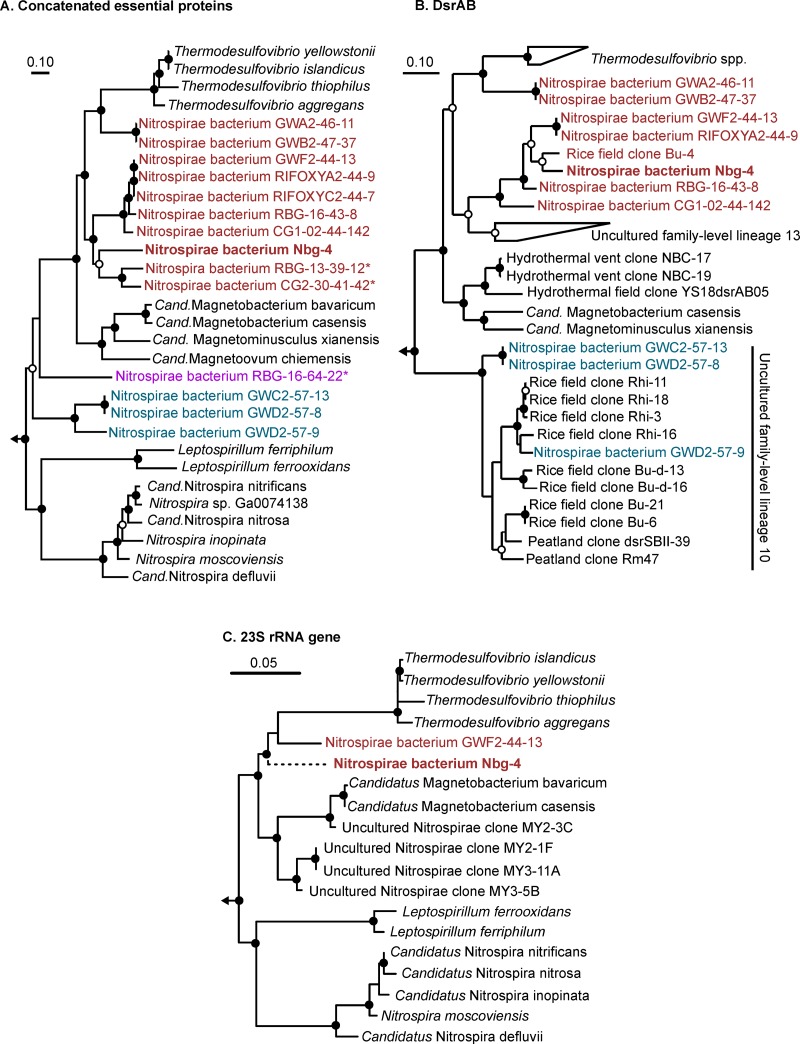
Phylogeny of Nitrospirae bacterium Nbg-4 (in boldface) and related *dsrAB*-carrying Nitrospirae bacteria recovered from metagenomes of groundwater systems (45, 46). Uncultured *dsrAB*-carrying Nitrospirae bacteria that form separate genera, as inferred by the genome-wide AAI approach, are color coded. Maximum-likelihood trees were inferred using the RAxML algorithm (79) and either a concatenated alignment of 43 essential proteins (67) (A), deduced DsrAB sequences (B), or the 23S rRNA gene (C). The partially recovered 23S rRNA gene of Nbg-4 was added to an RAxML tree of almost full-length 23S rRNA genes using the Quick add parsimony tool as implemented in ARB (82) without changing the tree topology. This is indicated by the dashed branch leading to Nbg-4 in this tree. Bootstrap support is indicated by filled (≥90%) and open (≥70%) circles at the respective branching points. The bars indicate 10% or 5% estimated sequence divergence.

A phylogenomic maximum likelihood tree placed Nbg-4 and 8 of the 14 *dsrAB*-carrying Nitrospirae bacteria recovered in other studies (Table S3 in the supplemental material) in a stable cluster that branched off between Thermodesulfovibrio spp. and magnetotactic Nitrospirae. Two additional *dsrAB*-carrying Nitrospirae bacteria (GWA2-46-11 and GWB2-47-37) formed a sister branch to the Nbg-4-containing cluster and were more closely related to Thermodesulfovibrio species (Fig. 4A). The remaining four *dsrAB*-carrying Nitrospirae bacteria branched off more basally within the phylum Nitrospirae, forming two separate lineages with no clear affiliation to previously isolated species (Fig. 4A).

The same branching pattern was recovered when deduced DsrAB sequences were analyzed. Here, the well separated Nbg-4-containing cluster was most closely related to uncultured DsrAB family-level lineage 13 as defined by A. L. Müller et al. (37). These two clusters shared a common origin branching off between Thermodesulfovibrio species and magnetotactic Nitrospirae (Fig. 4B). As with the phylogenomics approach, Nitrospirae bacteria GWA2-46-11 and GWB2-47-37 formed a stable sister branch that was more closely related to Thermodesulfovibrio species. Interestingly, the DsrAB proteins of Nitrospirae bacterium RBG-13-39-12 and CG2-30-41-42, which were the closest relatives to Nbg-4 by the phylogenomics approach, did not fall into the Nitrospirae supercluster but were most closely related to uncultured DsrAB family-level lineage 11, which belongs to the Deltaproteobacteria supercluster (see Fig. S1 in the supplemental material). This indicates lateral transfer of *dsrAB* within the phylum Nitrospirae, which is further supported by the DsrAB phylogeny of the basally branching Nitrospirae bacterium RBG-16-64-22. Here, the respective DsrAB sequences were clearly affiliated with the oxidative bacterial-type DsrAB, having the alphaproteobacterium Magnetococcus marinus and Chlorobi spp. as closest relatives (Fig. S1). In contrast, DsrAB of Nitrospirae bacteria that formed the second basally branching lineage by the phylogenomics approach were also clustering basally in the DsrAB Nitrospirae supercluster; they clustered within, or as the closest relatives to, uncultured DsrAB family-level lineage 10 (Fig. 4B).

In a third approach, the phylogenetic position of the partial 23S rRNA gene of Nbg-4 was inferred when it was placed into a full-length 23S rRNA gene tree of cultured and uncultured members of the phylum Nitrospirae. Here also, Nbg-4 branched off between stable clusters related to Thermodesulfovibrio species and magnetotactic Nitrospirae (Fig. 4C), corroborating the phylogenetic placement of the other two approaches.

In parallel, genome-wide average nucleotide identity (gANI) and average amino acid identity (gAAI) analyses were performed (48–50). The gANI analysis revealed that all Nitrospirae genomes used for the phylogenomic tree reconstruction were <70% similar to the genome of Nbg-4 (see Table S4 in the supplemental material). Since this is well below the proposed value of 96.5% for grouping bacterial strains into the same species (49), Nbg-4 represents a novel species. The gAAI analysis mainly mirrored the phylogenomic tree reconstruction. Here, all genomes within the Nbg-4-containing cluster, as well as the sister branch that encompasses *dsrAB*-carrying Nitrospirae bacteria GWA2-46-11 and GWB2-47-37, shared identities between 55 and 100% (see Table S5 in the supplemental material). At the same time, these genomes shared <55% identity with representatives of other genera within the phylum Nitrospirae. In addition, the two basally branching lineages of *dsrAB*-carrying Nitrospirae genome bins represented either by Nitrospirae bacterium RBG-16-64-22 or by Nitrospirae bacteria GWC2-57-13, GWD2-57-8, and GWD2-57-9 shared <55% gAAI identity to Nitrospirae spp. outside their respective lineages. At the same time, the latter three *dsrAB*-carrying Nitrospirae bacteria shared among themselves gAAI identities of 62 to 99% (Table S5). Since 55% gAAI is the lower boundary that is currently recommended for grouping bacterial strains into the same genus (48), Nbg-4 and the additional uncultured *dsrAB*-carrying Nitrospirae bacteria listed in Table S3 form three independent genera.

## DISCUSSION

Members of the phylum Nitrospirae that form a stable clade between thermophilic Thermodesulfovibrio spp. and magnetotactic Nitrospirae are regularly observed in 16S rRNA gene- and *dsrAB*-based surveys of anoxic freshwater and marine environments of moderate temperature. These environments include marine (37) and estuarine (51) sediments, groundwater (45, 52), lake sediment (53), wetland soil (54), an anoxic bioreactor (55), and rice paddy fields (10, 56, 57). Also in rice paddy soil analyzed in this study, eight species-level operational taxonomic units (OTUs) of such Nitrospirae were observed previously by 16S rRNA gene-based amplicon sequencing (Fig. S2) (7). In this study, we presented a detailed genome analysis of Nitrospirae bacterium Nbg-4 as a representative of this clade and analyzed its protein expression profile under sulfate-enriched and sulfate-depleted conditions in planted rice paddy microcosms.

Nbg-4 encoded the complete pathway for dissimilatory sulfate reduction (Fig. 2). Indeed, there are several lines of evidence that this newly discovered member of the Nitrospirae could represent an active sulfate reducer in rice paddy soil. From a genomic perspective, Nbg-4 carries not only all the genes necessary for sulfate reduction but also genes of unknown function that are typically found in SRM, such as *dsrD*, *dsrN*, and *dsrT* (40). The same *dsr* operon organization (Fig. 3), as well as the presence of all sulfate reduction-related genes (Table 2), was observed in the genomes of the other *dsrAB*-carrying Nitrospirae bacteria that form a stable phylogenetic lineage with Nbg-4 (Fig. 4). From a phylogenetic perspective, DsrAB of Nbg-4 and related Nitrospirae bacteria were clearly affiliated with the branch of reductively operating DsrAB of bacterial origin, which are phylogenetically separated from oxidatively operating DsrAB of bacterial origin (37). Most importantly, peptides clearly belonging to enzymes involved in sulfate reduction were preferentially detected for Nbg-4 in gypsum-treated bulk soil, i.e., under completely anoxic and sulfate-enriched conditions. In contrast, under sulfate-depleted conditions in control bulk soil, peptides clearly belonging to the enzyme complex involved in the first step of DNRA were detected. From pure-culture SRM capable of DNRA, it is known that sulfate is preferentially respired even in the presence of the thermodynamically more favorable electron acceptor nitrate and that expression of DNRA-related enzymes is induced only in the absence of sulfate, which acts as a repressor (58).

Nevertheless, involvement of Nbg-4 and related *dsrAB*-carrying Nitrospirae in anaerobic sulfur oxidation cannot be ruled out. For example, a study conducted in parallel to ours reported recently on the enrichment of a novel Nitrospirae species in an anoxic bioreactor that operated under simultaneous sulfide, methane, and ammonium consumption at the expense of nitrate (55). This novel Nitrospirae species closely resembled Nbg-4 in its genomic and phylogenetic features; sulfide oxidation coupled to DNRA is one of several explanations of its enrichment (55). Also, dense cell suspensions of the SRM Desulfovibrio desulfuricans and Desulfobulbus propionicus are capable of coupling sulfide oxidation to nitrate reduction (59) and S^0^ oxidation to electron transfer to a graphite electrode (60), respectively. In addition, Desulfurivibrio alkaliphilus was recently shown to grow by sulfide oxidation coupled to DNRA while encoding and transcribing DsrAB affiliated with the phylogenetic branch of reductively operating sulfite reductases (41). D. alkaliphilus carried and also expressed all other genes of the canonical pathways of sulfate reduction while oxidizing sulfide coupled to DNRA. At the same time, it lacked all typical sulfur metabolism genes of chemolithotrophic sulfur oxidizers except for a membrane-bound sulfide-quinone oxidoreductase (Sqr). This led to the proposal that the canonical pathway of sulfate reduction could act in reverse when coupled to Sqr (41). Interestingly, Nbg-4 also encoded Sqr, which showed moderate similarity (54% amino acid identity) to D. alkaliphilus Sqr. However, Nbg-4 Sqr could not be demonstrated to be expressed in the rice paddy metaproteomes analyzed (Table S2). The overall picture is further complicated by the phylogenetic placement of Nbg-4 and related *dsrAB*-carrying Nitrospirae between the genus Thermodesulfovibrio, which contains exclusively sulfate-reducing species, and magnetotactic *dsrAB*-carrying Nitrospirae, which are proposed to be capable of sulfur oxidation. Since genes encoding the biosynthesis of magnetosomes were not detected in the largely recovered genome of Nbg-4, and Nbg-4 was significantly more abundant in the completely anoxic bulk soil (Fig. 1), a lifestyle comparable to that of magnetotactic Nitrospirae can most likely be excluded.

In a preceding study, exclusively members of the Deltaproteobacteria (Syntrophobacter, Desulfovibrio, unclassified Desulfobulbaceae, and unclassified Desulfobacteraceae species) were shown to respond by population increase to higher sulfate availability in rice paddy soil (7). The current study utilized soil from exactly the same experiment and identified Nbg-4 as an additional potential SRM. Nbg-4 did not respond to sulfate availability with changes in population size (Fig. 1) but most likely responded by a switch in energy metabolism, i.e., from nitrate reduction under sulfate-depleted conditions to sulfate reduction under sulfate-enriched conditions (see above). This interpretation is supported by porewater sulfate turnover in microcosms incubated in parallel to those analyzed in this study (7), where sulfate concentrations steadily declined from 2.6 to 0.5 mM throughout the incubation period in gypsum-amended bulk soil but were below the detection limit in unamended bulk soil. Together, both studies reveal that rice paddy SRM may follow different ecological strategies, either by an activity response coupled to growth (Deltaproteobacteria) or by switching the energy metabolism to maintain a stable population (Nbg-4). Interestingly, species-level OTUs obtained in the previous study, which fall into a phylogenetic lineage resembling the Nbg-4 cluster (Fig. S2), constituted populations with relative sizes of ≤0.2% of the overall bacterial community in bulk soil irrespective of gypsum treatment (reanalyzed from reference 7). This is clearly above the currently recognized threshold of the so-called “rare biosphere” (<0.1%) but below the threshold of dominating species (>1%) (61, 62). As such, these novel Nitrospirae constitute moderately abundant members of the bacterial bulk soil community. This is in agreement with a study of three different Chinese rice paddy soils, where comparable population sizes were recorded (56).

Nbg-4 and related *dsrAB*-carrying Nitrospirae, which were all recovered from groundwater systems, clearly formed a separate lineage within the Nitrospirae. This was supported by three independent phylogeny inference approaches based on highly conserved marker genes, the *dsrAB* genes, and the 23S rRNA gene (Fig. 4). Further indirect evidence was provided by the same branching pattern of 16S rRNA genes affiliated with the phylum Nitrospirae and recovered from the same microcosms (Fig. S2). In accordance with the gAAI analysis performed, Nbg-4 and related *dsrAB*-carrying Nitrospirae that form this separate lineage constitute a newly discovered genus (Table S5). In addition, on the basis of the gANI analysis performed, Nbg-4 represents a species clearly distinct from all other members of this novel genus (Table S4).

### Description of a new Candidatus genus and species.

Based on its distinct potential physiology, separation into its own phylogenetic lineage, and its predominant occurrence in habitats of moderate temperature, the following name is proposed for Nbg-4: “Candidatus Sulfobium mesophilum,” gen. nov., sp. nov. (Sul.fo′bi.um. L. n. *sulfur*, sulfur; Gr. n. *bios*, life; N.L. neut. n. Sulfobium, a living entity metabolizing sulfur compounds; me.so′phi.lum. Gr. adj. *mesos*, middle; Gr. adj. *philos*, friend, loving; N.L. neut. n. mesophilum, loving medium temperatures). “Candidatus Sulfobium mesophilum” encodes the complete pathways for dissimilatory sulfate reduction and nitrate reduction to ammonia. Based on its genome, it is able to utilize butyrate, acetate, formate, and molecular hydrogen as electron donors. With a complete Wood-Ljungdahl pathway, “Candidatus Sulfobium mesophilum” possesses the metabolic potential to oxidize organic matter completely to CO_2_.

## MATERIALS AND METHODS

### Rice paddy microcosms.

Soil from planted rice paddy microcosms described by Wörner et al. (7) was analyzed. In brief, microcosms were sampled destructively after 58 to 59 days of greenhouse incubation (late vegetative phase of rice plants) to obtain rhizosphere and bulk soil samples of microcosms that were either left untreated (control) or treated with gypsum (0.15% [wt/wt] CaSO_4_·2H_2_O). In addition, freshly flooded soil was incubated for 3 days in the absence of rice seedlings and was designated T_0_. As such, the experimental setup resulted in five different soil habitats: bulk soil with or without gypsum addition, rhizosphere soil with or without gypsum addition, and freshly flooded soil. Sampling from the different soil compartments and DNA extraction based on bead beating and phenol-chloroform extraction were carried out as described by Wörner et al. (7).

### Metagenome sequencing, assembly, and binning.

Rhizosphere- and bulk soil-derived DNA extracts were obtained from four separate microcosms per treatment (gypsum and control). In addition, three DNA samples were obtained from freshly flooded soil. For each replicate, 2 μg of DNA was used for metagenomic library preparation and paired-end sequencing (2 × 100 bp) on an Illumina HiSeq 2000 platform at the King Abdullah University of Science and Technology, Thuwal, Saudi Arabia. Raw reads were processed in the CLC Genomics Workbench, v.5.5.1 (CLC bio, Aarhus, Denmark) using only paired-end reads of >50 bp with ≤1 ambiguous base calls and a quality score of ≥0.03 (corresponding to 99% accuracy). *De novo* assembly of pooled reads per habitat type was done in CLC using a k-mer size of 41 (determined as optimal in preliminary tests). Contigs with <2,000 bp were discarded. Scaffolds containing 16S rRNA genes, 23S rRNA genes, or *dsrAB* were identified by a blastn search (63) against the respective SILVA reference databases, v.123 (64), or a *dsrAB* reference database (37). Coverage of scaffolds was determined in CLC using 100% identity over the full length of quality-trimmed reads. This was done for each sequenced replicate separately for statistical analysis and, in addition, by using pooled replicates per habitat type for genome binning.

Genome binning was performed according to the method of Albertsen et al. (65) using the gypsum and control treatments as differential coverage conditions (see Fig. S3 in the supplemental material). From the 159 genome bins obtained, a *dsrAB*-carrying Nitrospirae bin assembled from gypsum-treated bulk soil was selected for further refinement (Fig. S3). First, quality-trimmed reads that mapped to the Nitrospirae bin as well as to taxonomically unaffiliated scaffolds of similar coverage were reassembled in CLC and were binned as outlined above. Thereafter, the scaffolds obtained were coassembled with quality-trimmed reads of the first step using SPAdes (66). Binning resulted in the genome bin Nbg-4 (*Nitrospirae* genome bin from bulk soil treated with gypsum). Using this procedure, the genome of Nbg-4 could be extended from 1.15 Mbp with 57 of 107 queried essential single-copy genes (ESGs) to 2.77 Mbp that covered 92 ESGs, 91 of which were present as one copy. Assembly refinement of a 23S rRNA gene fragment carried at the end of one Nbg-4 scaffold is described in the supplemental material. The completeness, contamination, and strain heterogeneity of Nbg-4 were evaluated using CheckM (67). To assess its relative abundances in the different soil habitats, quality-trimmed reads of sequenced soil replicates were mapped with a similarity threshold of 100% over the complete read to the Nbg-4 scaffolds using CLC. Mapped reads were normalized to RPKM (reads per kilobase of scaffold per million reads) values.

### Annotation and additional analyses.

The MicroScope platform was used for automatic annotation (68, 69). Annotation refinement was done as follows. Proteins with an amino acid identity of ≥40% (over ≥80% of the sequence) with a Swiss-Prot entry (70) were annotated as homologous to proteins with a known function. Proteins with an amino acid identity of ≥25% (over ≥80% of the sequence) to a Swiss-Prot or TrEMBL (70) entry were annotated as putative homologs of the respective database entries.

Genome-wide average nucleotide identity (gANI) (50) and genome-wide average amino acid identity (gAAI) (48) comparisons were performed using the Web service of the Konstantinidis laboratory at the Georgia Institute of Technology, Atlanta, GA, USA (enve-omics.ce.gatech.edu). The index of replication (iRep) was calculated using the iRep software (39). SAM files needed as input for iRep were created using bowtie2 (71).

To estimate the effect of soil habitat, gypsum treatment, and their interaction on the relative abundance of the Nitrospirae genome bin, a two-way ANOVA was performed based on the RPKM values of its longest scaffold (106,945 bp) in the different replicated metagenomes. This was done using the base package of the R program, v.3.1.1 (72). Assumptions of variance homogeneity and normality were tested using Levene's test in the R package lawstat (73). Significant differences between differently treated soil habitat types were inferred using Tukey's test of honestly significant difference.

### Metaproteomics of rice paddy soils.

Total proteins were extracted from the same replicated soil samples as those used for metagenome sequencing. Protein extraction and subsequent in-gel tryptic digestion followed the procedure outlined by Starke et al. (74). Briefly, 2 g of soil was used for a phenol extraction procedure with subsequent ammonium acetate precipitation. Tryptic peptides were analyzed using an ultraperformance liquid chromatography (UPLC)-linear trap quadrupole (LTQ) Orbitrap Velos liquid chromatograph-tandem mass spectrometer (LC–MS-MS) (75). Peptide searches were performed using the MaxQuant algorithm with the following parameters: tryptic cleavage with a maximum of two missed cleavages, a peptide tolerance threshold of ±10 ppm, an MS-MS tolerance threshold of ±0.5 Da, and carbamido methylation at cysteines as static and oxidation of methionines as variable modifications. As a sample specific database, the Nbg-4 genome was used. According to currently accepted practice in metaproteomics (76, 77), proteins were considered to be identified with at least one unique peptide with high confidence (false-discovery-rate-corrected *P* value, <0.01). To check for false-positive assignments, all metaproteome replicates were also searched against the complete bacterial protein database of NCBI (August 2017).

### Phylogenetic analysis.

Additional Nitrospirae genome bins carrying *dsrAB* were identified using a blast search (63) against NCBI's sequence repositories (78). Only Nitrospirae genome bins with a completeness above 70% and a contamination level below 7% according to CheckM (67) were considered for further analysis. The phylogenetic affiliations of Nbg-4 and public *dsrAB*-carrying Nitrospirae genome bins were inferred by a phylogenomics approach based on 43 conserved marker genes with largely congruent phylogenetic histories as defined by Parks et al. (67), or using the *dsrAB* and 23S rRNA genes as phylogenetic markers. The respective maximum likelihood trees were calculated using RAxML, v.8.2.9 (79), as implemented on the CIPRES Web server (80) (www.phylo.org). Details are provided in the supplemental material.

### Accession number(s).

All metagenome sequences are available in the Sequence Read Archive of NCBI under BioProject number PRJNA391190. The draft genome of Nbg-4 has been deposited in EMBL under study accession number PRJEB21584. The mass spectrometry data have been deposited to the ProteomeXchange Consortium via the PRIDE partner repository (81) with the data set identifier PXD007817.

## Supplementary Material

Supplemental material
