# Supplementary material for: Rice Paddy Nitrospirae Carry and Express Genes Related to Sulfate Respiration: Proposal of the New Genus “Candidatus Sulfobium”
Source: Appl Environ Microbiol. 2018 Feb 14;84(5):e02224-17. doi: 10.1128/AEM.02224-17 (PMC5812927; doi:10.1128/AEM.02224-17)
Supplement: Supplemental material [file supp_84_5_e02224-17__index.html]

Supplemental material 

# Rice Paddy Nitrospirae Carry and Express Genes Related to Sulfate Respiration: Proposal of the New Genus “Candidatus Sulfobium”

## Supplemental material

- Supplemental file 1 -

  Supplemental materials and methods; phylogeny of deduced DsrAB sequences of *Nitrospirae* bacterium Nbg‐4 and related *dsrAB*-carrying *Nitrospirae* bacteria recovered from metagenomes of groundwater systems (Fig. S1); maximum likelihood 16S rRNA gene tree (Fig. S2); schematic overview of the bioinformatics workflow (Fig. S3).

  PDF, 530K
- Supplemental file 2 -

  Key characteristics of sequenced metagenomes (Table S1); annotation and locus of genes involved in energy and biosynthesis metabolism in *Nitrospirae* bacterium Ngb-4 (Table S2); main characteristics of members of the phylum *Nitrospirae* (Table S3); genome-wide average nucleotide (Table S4) and amino acid (Table S5) identity of *Nitrospirae* bacterium Nbg-4 in comparison to other members of the phylum *Nitrospirae*.

  XLS, 115K
