# Supplementary material for: Rice Paddy Nitrospirae Carry and Express Genes Related to Sulfate Respiration: Proposal of the New Genus “Candidatus Sulfobium”
Source: Appl Environ Microbiol. 2018 Feb 14;84(5):e02224-17. doi: 10.1128/AEM.02224-17 (PMC5812927; doi:10.1128/AEM.02224-17)
Supplement: Supplemental material [file AEM.02224-17_zam005188339s1.pdf]

Supplementary information for

Rice paddy *Nitrospirae* encode and express genes related to sulfate respiration: proposal of the new genus *Candidatus* Sulfobium

Sarah Zecchin*[a,b], Ralf C. Mueller*[a], Jana Seifert[c], Ulrich Stingl[d], Karthik Anantharaman[e], Martin von Bergen[f], Lucia Cavalca[b], Michael Pester[#a,g]

*contributed equally

[#]corresponding author

[a] *Department of Biology, University of Konstanz, Konstanz, Germany*

[b] *Dipartimento di Scienze per gli Alimenti, la Nutrizione e l'Ambiente (DeFENS), Università degli Studi di Milano, Milano, Italy*

[c] *Institute of Animal Science, Hohenheim University, Stuttgart, Germany*

[d] *University of Florida, UF/IFAS, Department for Microbiology & Cell Science, Fort Lauderdale Research and Education Center, Davie, FL, USA*

[e] *Department of Earth and Planetary Science, University of California, Berkeley, CA, USA*

[f] *Helmholtz Centre for Environmental Research—UFZ, Department of Molecular Systems Biology, Leipzig, Germany*

[g] *Department Microorganisms, Leibniz Institute DSMZ – German Collection of Microorganisms and Cell Cultures, Braunschweig, Germany*

Supplementary materials and methods

Assembly extension of the 23S rRNA gene of *Nitrospirae* bin Nbg-4

One of the *Nitrospirae* bin scaffolds contained a partial 23S rRNA gene of 89 bp at one of its ends. In an attempt to recover a longer fragment of this rRNA coding region, we mapped all quality-trimmed Illumina reads obtained from bulk soil treated with gypsum to the rrn operon of *Nitrospira defluvii* with a similarity threshold at or above 80% over the complete read length using CLC. The 138,750 reads that mapped to this operon were assembled with the SPAdes assembler (1) and resulted in a 693 bp-long contig, with its last 37 bp overlapping with 100% identity with the end of the partial

Nitrospirae 23S rRNA gene. Using this approach, the *Nitrospirae* 23S rRNA gene was elongated at its 3'-end from 89 to 745 bp. Thereafter, the completeness, contamination and strain heterogeneity of the obtained draft genome were evaluated using CheckM (2). This genome bin is referred to as Nbg-4 (***N****itrospirae* genome bin from **b**ulk soil treated with **g**ypsum) throughout the rest of the manuscript.

Phylogenetic analysis of the *Nitrospirae* draft genome

Additional *Nitrospirae* genome bins carrying *dsrAB* were identified using a blast search (3) against NCBI's sequence repositories (4). Only *Nitrospirae* genome bins with a completeness above 70% and a contamination below 7% according to CheckM (2) were considered for further analysis. The phylogenetic affiliation of Nbg-4 and public *dsrAB*-carrying *Nitrospirae* genome bins was inferred using a phylogenomics approach. This approach was based on a concatenated alignment of deduced amino acid sequences of 43 conserved marker genes with a largely congruent phylogenetic history (2). These marker genes are used by CheckM (2) for the placement of a query genome within a reference genome tree and their concatenated alignment is given as a general output of this program. Using this alignment, a maximum likelihood (ML) tree was inferred from 5,793 unambiguously aligned amino acid positions based on the Dayhoff substitution matrix and a gamma distribution model of substitution rate heterogeneity. The ML tree was calculated using RAxML v8.2.9 (5) as implemented on the CIPRES webserver (6, www.phylo.org). A rapid bootstrap analysis was performed by RAxML using the automatic MRE-based bootstrapping criterion (extended majority-rule consensus tree criterion) and stopped after 150 replicates. The tree was based on sequences retrieved from the following genome sequences (NCBI accession numbers): *Nitrospirae* (NZ_BCNO01000001, AXWU01000000, AUIU01000000, NC_011296, NC_018649, NC_017094, NZ_CP011801, LNDU01000000, JMFO01000000, LACI01000000, JZJI01000000, LNQR01000000, NC_014355, NZ_LN885086, CZPZ01000000, CZQA01000000, MHEJ01000000, MHEE01000000, MHEW01000000, MHDU01000000, MHDT01000000, MHEF01000000, MNVK01000000, MHEW00000000, MHEY00000000, MNYU00000000, MHEK00000000, MHDZ00000000, MHEC00000000, MHED00000000), Acidobacteria (NZ_JQKI00000000, NC_015064, NC_014963, NC_008536, NZ_AUAU00000000, NZ_AGSB00000000) and Deltaproteobacteria (NC_014972, NC_012108, NC_015388, NC_013173, NC_002937).

The phylogeny inferred by the phylogenomics approach was compared to the phylogenetic affiliation of the encoded *dsrAB* and partial 23S rRNA genes. For phylogenetic inference of deduced DsrAB amino acid sequences, insertions and deletions were removed from the dataset using an alignment mask (indel filter). Based on this alignment, a RAxML tree was inferred from 530 unambiguously aligned amino acid positions using the parameters outlined above. MRE-based bootstrap analysis stopped after 354 replicates. The tree was based on the following *dsrAB* sequences (NCBI accession

numbers): Nitrospirae supercluster (AF334599, U58122, EF429274, EF429277, EF429278, AUIU01000015, AB124925, AB124928, JN615155, AB451528, JN615146, JN615156, JN615158, JN615159, JN615164, JN615167, JN615169, JN615173, AY167472, GU127969, KF896967, KF896981, KF896982, EF065021, AB451527, MNVK01000061, MHDT01000018, MHDU01000003, MHEE01000015, LNQR01000029, MHEJ01000097, MHEW01000017, MHDZ01000017, MHEC01000106, MHED01000223, JMFO00000000), Deltaproteobacteria supercluster including laterally acquired-*dsrAB* Firmicutes (AB061535, AF271770, AF273030, AF074396, AF271769, NC_007644, AY626025, NC_010424, CP001785, CP001720, JQ304755, CP003273, AUBR01000022, JMFO00000000, AF482455, AM236170, EF065046, AM408825, AF551758, AF191907, AF482463, FO203503, DQ386236, JQ519394, JQ519395, AY083030, AY167475, EF065068, CP003360, AF418189, JQ519396, AB061539, AB061541, CP000112), and environmental supercluster (DQ112192, AY167483, GU371960, GU372072, KF896934, KF896940). Sequences not belonging to the *Nitrospirae* supercluster were used as outgroup. The same settings were used to evaluate lateral gene transfer of *dsrAB* within the phylum Nitrospirae (Fig. S3). MRE-based bootstrap analysis stopped after 252 replicates in this tree reconstruction.

For the phylogenetic inference of the partial 23S rRNA gene, a RAxML tree was initially calculated based on the nucleic acid alignment of almost full-length 23S rRNA gene sequences of cultured and environmental *Nitrospirae*. This was based on the alignment of the non-redundant 23S rRNA gene database v.123 available on the SILVA online platform (7, www.arb-silva.de) and a 50% conservation filter of nucleic acid positions within the phylum *Nitrospirae*. Based on this alignment, a RAxML tree was inferred from 2,732 unambiguously aligned nucleic acid positions based on the GTRGAMMA distribution model of substitution rate heterogeneity. MRE-based bootstrap analysis stopped after 156 replicates. The outgroup consisted of microorganisms belonging to the Actinobacteria (*Patulibacter medicamentivorans*, *Patulibacter minatonensis*, *Conexibacter woesei*, and *Rubrobacter xylanophilus*). The tree was based on the following 23S rRNA gene sequences (NCBI accession numbers): *Nitrospirae* (BBCX01000008, AXWU01000024, AUIU01000004, CP001147, CP002919, AP012342, CP011801, JMFO01000010, FP929063, FP929003, LN885086, CZPZ01000003, CZQA01000015, MHEE01000002) and Actinobacteria (AGUD01000068, JAFH01000035, AUKG01000002, CP000386). Actinobacteria were used as outgroup. The partial 23S rRNA gene of the Nbg-4 was added to this tree using the Quick add parsimony tool as implemented in ARB (8) without changing the tree topology.

Partial 16S rRNA reads obtained in a previous 454 amplicon sequencing of the same soil samples (9) were phylogenetically analyzed in ARB (8). Only reads representing an OTU at 97% sequence identity with more than 300 bp in length and affiliated to the phylum *Nitrospirae* were considered.

An initial RAxML tree was calculated based on the nucleic acid alignment of almost full-length 16S rRNA gene sequences of cultured and environmental *Nitrospirae* with acidobacterial 16S rRNA sequences serving as outgroup. The tree inference was based on an alignment of the non-redundant SILVA 16S rRNA gene database v.128 (7, www.arb-silva.de) and a 50% conservation filter of nucleic acid positions within the domain Bacteria (1,222 unambiguously aligned nucleic acid positions). The partial 16S rRNA genes of 454 read OTUs were added to this tree using the Quick add parsimony tool as implemented in ARB (8) without changing the tree topology.

Supplementary figures

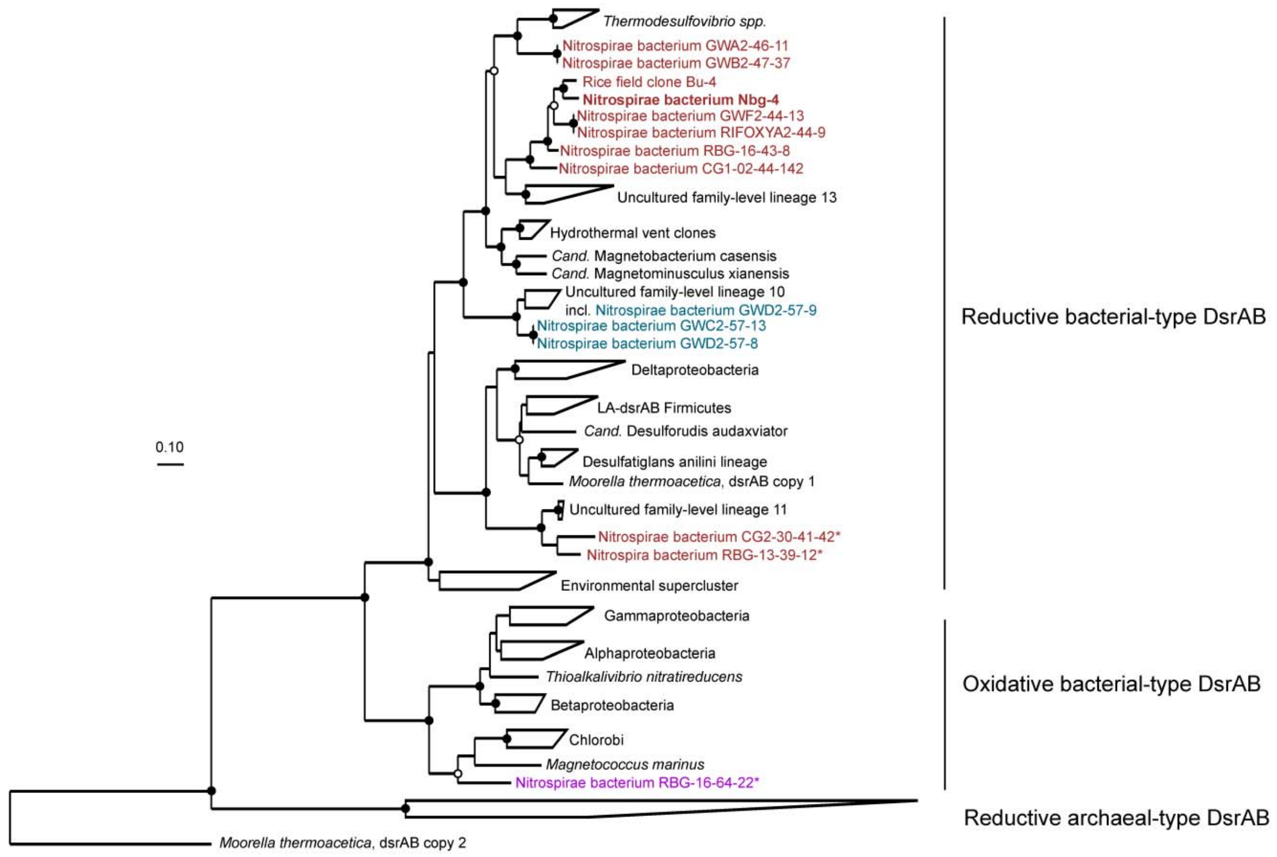

Figure S1. Phylogeny of deduced DsrAB sequences of *Nitrospirae* bacterium Nbg-4 and related *dsrAB*-carrying *Nitrospirae* bacteria recovered from metagenomes of groundwater systems (10, 11). A maximum likelihood tree were inferred using the RAxML algorithm (5). Bootstrap support is indicated by closed (≥90%) and open (≥70%) circles at the respective branching points. *Nitrospirae* bacteria with *dsrAB* that underwent horizontal gene transfer are marked with an asterisk. The scale bar indicates 10% estimated sequence divergence.

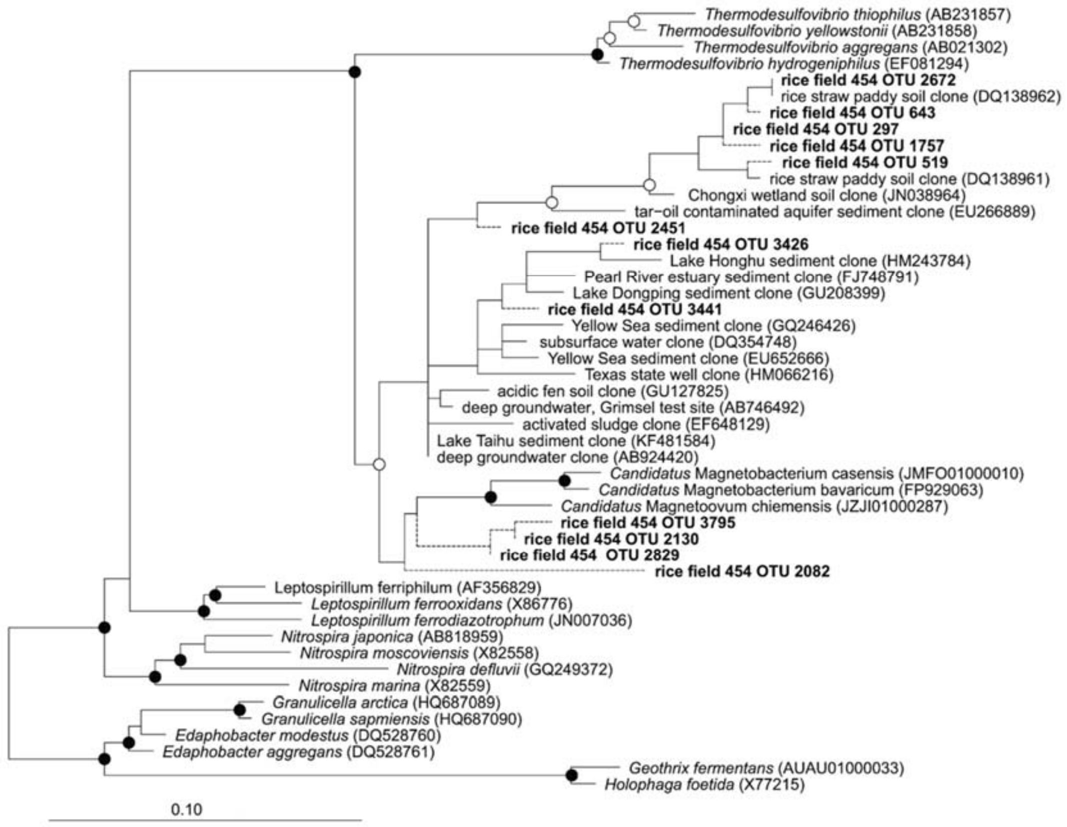

Figure S2. Maximum likelihood 16S rRNA gene tree showing the phylogenetic position of species-level OTUs affiliated to the phylum *Nitrospirae*, which were obtained in a previous study (9) using the same rice paddy soil samples as analyzed in the current study. The tree was reconstructed using the RAxML algorithm (5) as implemented in ARB (8) using 1,222 unambiguously aligned nucleotide positions and a 50% conservation filter for the domain Bacteria. The representative 454 amplicon sequences were added to the tree by using ARB's Parsimony Interactive tool as indicated by the dashed branch. Solid circles indicate ≥90% and open circles ≥70% bootstrap support (1000 replications). The bar represents 10% inferred sequence divergence.

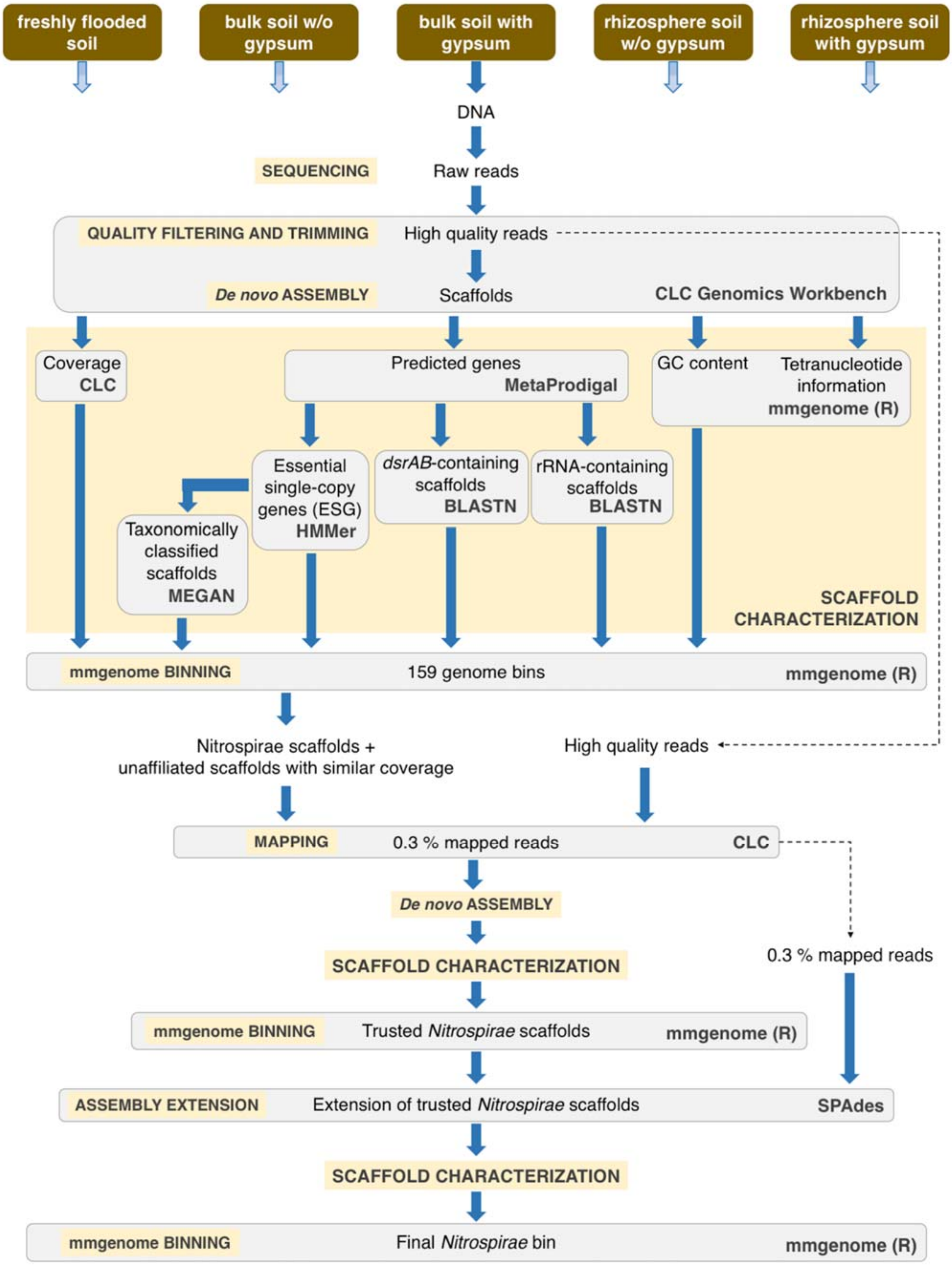

Figure S3. Schematic overview of the bioinformatics workflow to obtain the high quality draft genome of *Nitrospirae* bacterium Nbg-4.

Supplementary tables

Supplementary Tables S1-S5 are provided in a separate Excel file.

Supplementary references

1. Bankevich A, Nurk S, Antipov D, Gurevich AA, Dvorkin M, Kulikov AS, Lesin VM, Nikolenko SI, Pham S, Prjibelski AD, Pyshkin AV, Sirotkin AV, Vyahhi N, Tesler G, Alekseyev MA, Pevzner PA. 2012. SPAdes: a new genome assembly algorithm and its applications to single-cell sequencing. Journal of Computational Biology 19:455-477.
2. Parks DH, Imelfort M, Skennerton CT, Hugenholtz P, Tyson GW. 2015. CheckM: assessing the quality of microbial genomes recovered from isolates, single cells, and metagenomes. Genome Research 25:1043-1055.
3. Altschul SF, Gish W, Miller W, Myers EW, Lipman DJ. 1990. Basic local alignment search tool. Journal of Molecular Biology 215:403–410.
4. NCBI-Resource-Coordinators. 2017. Database resources of the National Center for Biotechnology Information. Nucleic Acids Research 45:D12-D17.
5. Stamatakis A. 2014. RAxML Version 8: A tool for phylogenetic analysis and post-analysis of large phylogenies. Bioinformatics 30:1312–1313.
6. Miller MA, Pfeiffer W, Schwartz T. 2010. Creating the CIPRES Science Gateway for inference of large phylogenetic trees, abstr Gateway Computing Environments Workshop (GCE), New Orleans, LA, USA, 14 Nov. 2010
7. Quast C, Pruesse E, Yilmaz P, Gerken J, Schweer T, Yarza P, Peplies J, Glöckner FO. 2013. The SILVA ribosomal RNA gene database project: improved data processing and web-based tools. Nucleic Acids Research 41:D590-D596.
8. Ludwig W, Strunk O, Westram R, Richter L, Meier H, Yadhukumar, Buchner A, Lai T, Steppi S, Jobb G, Forster W, Brettske I, Gerber S, Ginhart AW, Gross O, Grumann S, Hermann S, Jost R, Konig A, Liss T, Lussmann R, May M, Nonhoff B, Reichel B, Strehlow R, Stamatakis A, Stuckmann N, Vilbig A, Lenke M, Ludwig T, Bode A, Schleifer K-H. 2004. ARB: a software environment for sequence data. Nucl Acids Res 32:1363–1371.
9. Wörner S, Zecchin S, Dan J, Todorova NH, Loy A, Conrad R, Pester M. 2016. Gypsum amendment to rice paddy soil stimulated bacteria involved in sulfur cycling but largely preserved the phylogenetic composition of the total bacterial community. Environmental

Microbiology Reports 8:413–423.
10. Anantharaman K, Brown CT, Hug LA, Sharon I, Castelle CJ, Probst AJ, Thomas BC, Singh A, Wilkins MJ, Karaoz U, Brodie EL, Williams KH, Hubbard SS, Banfield JF. 2016. Thousands of microbial genomes shed light on interconnected biogeochemical processes in an aquifer system. Nature Communications 7:13219.
11. Probst AJ, Castelle CJ, Singh A, Brown CT, Anantharaman K, Sharon I, Hug LA, Burstein D, Emerson JB, Thomas BC, Banfield JF. 2016. Genomic resolution of a cold subsurface aquifer community provides metabolic insights for novel microbes adapted to high CO_2 concentrations. Environmental Microbiology 19:459–474.
